# Editorial: Immune regulation in sepsis

**DOI:** 10.3389/fimmu.2023.1298777

**Published:** 2023-10-06

**Authors:** Huiling Zhang, Zuliang Jie, Peisong Gao, Yufeng Zhou, Duanwu Zhang

**Affiliations:** ^1^ Children’s Hospital of Fudan University, National Children’s Medical Center, and Shanghai Key Laboratory of Medical Epigenetics, International Co-laboratory of Medical Epigenetics and Metabolism, Ministry of Science and Technology, Institutes of Biomedical Sciences, Fudan University, Shanghai, China; ^2^ State Key Laboratory of Cellular Stress Biology, School of Life Sciences, Faculty of Medicine and Life Sciences, Xiamen University, Xiamen, China; ^3^ Division of Allergy and Clinical Immunology, Johns Hopkins University School of Medicine, Baltimore, MD, United States

**Keywords:** immune regulation, sepsis, inflammation, immunosuppression, macrophage, neutrophil

Sepsis leads to high morbidity and mortality worldwide without effective treatments. Patients with severe sepsis often develop symptoms of persistent inflammation, immunosuppression, and catabolic syndrome involving multiple cell types, organ systems, and pathophysiological mechanisms. Mechanistically, the immune response initiated by an invading pathogen fails to return to normal homeostasis, leading to sustained excessive inflammation and immune suppression in sepsis (1). Sepsis-associated excessive inflammation is prominently featured in leukocytes (i.e., neutrophils, macrophages, natural killer cells), endothelial cells, cytokines, complement products, and the coagulation system, while immunosuppression is related to impaired functions of immune cells (e.g., exhaustion of T cells, apoptosis of T cells, B cells and dendritic cells, reduced human leukocyte antigen-DR isotype (HLA-DR) expression by antigen-presenting cells), diminished production of proinflammatory cytokines through epigenetic modification and increased release of anti-inflammatory cytokines (2).

The goal of this Research Topic is to provide a forum to advance research on the contribution of immune system to the development and progression of sepsis. We seek to study the molecular mechanisms of cytokine release syndrome and immunosuppression, develop biomarkers for early diagnosis and prognosis, and explore innovative immunopharmacological interventions to treat sepsis.

We have received several original articles that report new targets for curing sepsis. Two of them concerned neutrophil infiltration and function. Ni et al. identified Fpr2 as a negative regulator of Streptococcus suis-induced streptococcal toxic shock-like syndrome (STSLS). They found that *Fpr2*-knockout mice were less susceptible to STSLS with increased neutrophil recruitment but without an impact on neutrophil extracellular trap (NET) construction. Koutsogiannaki et al. defined αDβ2 as a novel potential target of sepsis. They reported that αDβ2 deficiency attenuated lung injury and improved survival in experimental polymicrobial abdominal sepsis. Further analyses showed that αDβ2 could increase phagocytosis and reduce apoptosis of neutrophils.

Two groups focused on the regulation of macrophage inflammation in sepsis. Wang et al. explored the effect of S1PR3 on NLRP3 inflammasome activation and sepsis mortality. They observed that one of the five S1PRs, S1PR3, was upregulated in macrophages in the inflammatory state. Inhibition of S1PR3 significantly suppressed ATP-induced NLRP3 inflammasome activation in macrophages. Mechanistically, inhibition of S1PR3 suppressed the LPS priming signal and reduced TWIK2 membrane trafficking-mediated potassium efflux. However, the S1PR3 antagonist increased mouse mortality in polymicrobial sepsis, suggesting the complexity between NLRP3 inflammasome activation and sepsis burden. Circular RNAs (circRNAs) have been linked to the regulation of macrophage polarization and subsequent inflammation in sepsis. Yang et al. built a sepsis circRNA expression pool and verified circ_0075723 as one of the markedly downregulated circRNAs, which might be related to pyroptosis. They detected the pyroptosis-associated signature in sepsis patients and linked pyroptosis with sepsis. circ_0075723 could inhibit pyroptosis by functioning as a sponge for miR-155-5p and regulating SHIP1 expression in macrophages. This work proposed that circ_0075723 could be a novel target for treating pneumonia-induced sepsis.

Vascular endothelial cells are the first cells to sense the damage induced by sepsis. Liu et al. identified an important role of Notch1 in maintaining vascular permeability during sepsis. Sepsis mediators downregulated the intracellular domain of Notch1 (NICD) and downstream signaling, further impairing endothelial barrier function. NICD overexpression and agonist stimulation alleviated endothelial dysfunction under inflammatory conditions *in vitro* and elevated the survival rate of septic mice *in vivo*. This study proposed the Notch1-Akt axis as a potential therapeutic target for sepsis. In line with this work, Ying et al. showed that circulating SDC1 levels were persistently upregulated in children with septic shock and that SDC1 shedding caused endothelial permeability under septic conditions. They found that the drug sulodexide could reduce endothelial permeability by preventing SDC1 shedding. Sulodexide administration alleviated lung injury and restored endothelial glycocalyx damage.

Cytokine signaling participates in the defense against pathogens. Wang et al. reported that γδT cell-intrinsic IL-1R signaling promoted survival during *Staphylococcus aureus* bacteremia. Using several systematic and conditional knockout mice, the authors found that IL-1R signaling in γδT cells (but not in other immune cells) was involved in the defense against bacterial infection by promoting monocyte recruitment.

Acute liver injury (ALI) is common in sepsis patients and is significantly associated with poor prognosis. Cai et al. investigated the roles and mechanisms of mesenchymal stem cells (MSCs) in treating ALI in sepsis. They found that either MSCs or exosome extracted from MSCs could attenuate ALI and subsequent death in sepsis. Further research showed that miR-26a-5p in MSC-derived exosome protected against hepatocyte death and liver injury caused by sepsis by targeting MALAT1. Therefore, they proposed miR-26a-5p/MALAT1 as novel targets for drug development in treating ALI in sepsis.

Two research articles focused on finding new prognostic markers for sepsis. Two markers were incidentally related to neutrophil infiltration. Liang et al. developed a new sepsis prognostic risk system. They first identified pyroptosis-related DEGs between sepsis patients and healthy cohorts, then narrowed down the field through functional analysis and immune infiltration analysis and constructed the prognostic risk system based on six pyroptosis-related genes (*GZMB*, *CHMP7*, *NLRP1*, *MYD88*, *ELANE*, and *AIM2*), which were all associated with neutrophils. Four of the six genes (*GZMB*, *CHMP7*, *NLRP1*, and *AIM2*) had potential diagnostic value in sepsis diagnosis. Preechanukul et al. utilized a reductionist approach leveraging publicly available transcriptomic data to explore effective risk and outcome predictors in sepsis. They searched for upregulated genes by *in vitro* exposure of neutrophils from healthy subjects with the serum of septic patients and identified *ACVR1B* as a candidate. *ACVR1B* expression was shown to be upregulated in septic melioidosis and could be a prognostic marker of sepsis.

The imbalance of pro-inflammation and anti-inflammation can lead to sepsis. FES tyrosine kinase is highly expressed in innate immune cells and plays a role in limiting the overactivation of the immune system. Laight et al. studied whether enhancing the expression of FES in early sepsis and inhibiting its effects in late sepsis could improve outcomes. They employed *in vivo*, *in vitro*, and clinical research methodologies to elucidate the role of FES in sepsis. Cell types, different sepsis models, agonists and antagonists, and timing of FES regulation were included in their research scope to accurately evaluate the role of FES in sepsis.

Gut motility dysfunction is the most common complication of post-septic organ dysfunction, which involves immune and neuronal cells. Yao et al. observed that muscular neutrophil infiltration leading to neuron loss in the intestinal muscle caused intestinal motility dysfunction after pneumonia sepsis. However, neutrophil blockade was ineffective in relieving sepsis. The authors found that MHCII^hi^ CX3CR1^hi^ macrophages could recover intestinal motility in pneumonia sepsis depending on prostaglandin E2 (PGE2). This work provides more insights into intestinal dysmotility after sepsis.

Immunoparalysis, an immunosuppressed state, is associated with worsened outcomes, including multiple organ dysfunction syndrome, secondary infections, and increased mortality. Joshi et al. reviewed that low human leukocyte antigen-DR isotype (HLA-DR) expression on peripheral blood monocytes was correlated with increased risks for infection and death. In particular, they emphasized that sargramostim, a recombinant human granulocyte-macrophage colony-stimulating factor (rhu GM-CSF), could stimulate and restore immune functions in sepsis immunoparalysis.

In summary, articles in this Research Topic highlight the critical roles of immune regulation in sepsis and are depicted in **Figure 1**. However, these insights into sepsis have yet to be translated into effective treatments for sepsis. Further investigations are urgently needed to better understand the immunopathogenesis of sepsis so that better prognostic markers and effective treatments can be developed.

**Figure 1:**
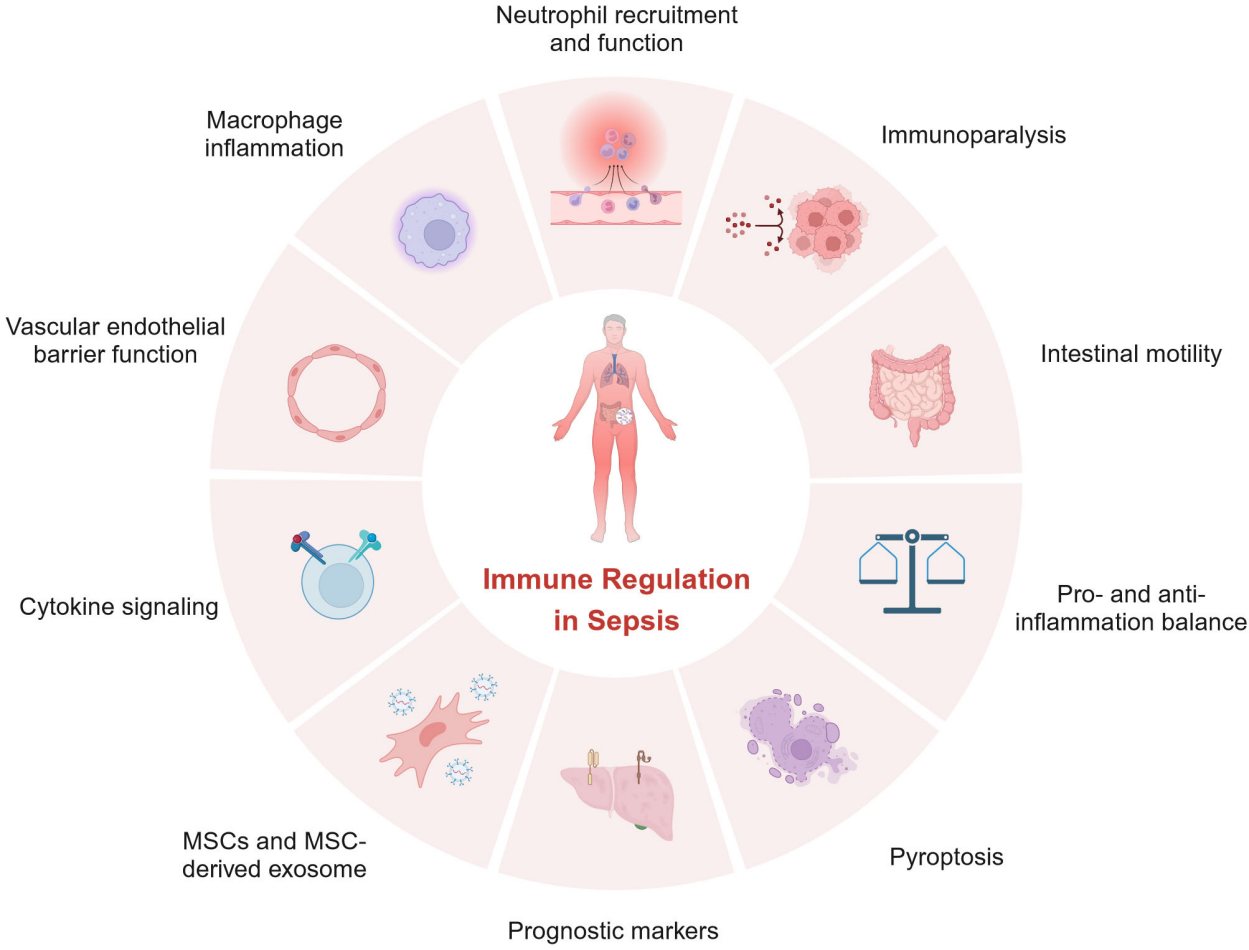
Perspectives of the Research Topic: Immune Regulation in Sepsis.

## Author contributions

HZ: Writing – original draft. ZJ: Writing – review & editing. PG: Writing – review & editing. YZ: Writing – review & editing. DZ: Funding acquisition, Writing – original draft, Writing – review & editing.
